# Embracing complexity: theory, cases and the future of bioethics

**DOI:** 10.1007/s40592-014-0001-z

**Published:** 2014-10-10

**Authors:** James Wilson

**Affiliations:** Department of Philosophy, University College London, Gower Street, London, WC1E 6BT UK

**Keywords:** Translational research, Ideal theory, Thought experiments, Empirical bioethics, Complexity, External validity

## Abstract

This paper reflects on the relationship between theory and practice in bioethics, by using various concepts drawn from debates on innovation in healthcare research—in particular debates around how best to connect up blue skies ‘basic’ research with practical innovations that can improve human lives. It argues that it is a mistake to assume that the most difficult and important questions in bioethics are the most abstract ones, and also a mistake to assume that getting clear about abstract cases will automatically be of much help in getting clear about more complex cases. It replaces this implicitly linear model with a more complex one that draws on the idea of translational research in healthcare. On the translational model, there is a continuum of cases from the most simple and abstract (thought experiments) to the most concrete and complex (real world cases). Insights need to travel in both directions along this continuum—from the more abstract to the more concrete *and* from the more concrete to the more abstract. The paper maps out some difficulties in moving from simpler to more complex cases, and in doing so makes recommendations about the future of bioethics.

## Introduction

Bioethics is often thought to be subject to two competing aims, which we can refer to as the theoretical and the practical. The theoretical aim is to arrive at correct (or at least well justified) answers to normative questions such as whether it is ever ethically acceptable for a doctor to prescribe a deceptive placebo to a patient, or whether hard paternalism is ever permissible. The practical aim is to bring the world closer to some normatively justified state of affairs—perhaps by making it easier to get compulsory licenses for essential medicines, or by implementing a particular policy to reduce health inequities.

The two aims seem to pull bioethicists in different directions, because it is plausible to think that the most effective means of pursuing the theoretical aim will be relatively ineffective in pursuing the practical aim, and vice versa. This apparent conflict arises much more strongly for bioethics than it does, say, for political philosophy. In part this is because the questions that bioethics deals with are often much more obviously shaped by our present concerns than those in political philosophy, and in part it is because there is no more applied academic discipline than bioethics that stands in the relationship to it that political science and political theory stand to political philosophy. In brief, we tend to approach bioethics with the goal of improving our grip on real world ethical questions, and in working out what we should do all things considered, the buck stops with it.

Perhaps the most significant reason for the lack of congruence between the theoretical and the practical aims is that public ethical discourse relies much more on narratives and systems of analogies than on rigorous normative arguments. Persuading others to think of a problem within a certain frame is usually much more practically effective than presenting a cogent normative argument that has no obvious connection to any existing policy frames or narratives.^1^ Overwhelmingly, the frame that is put on an issue determines the arguments that will have political currency, rather than vice versa. For this reason, much political debate consists in telling stories that encourage citizens to apply one rather than another pre-existing frame to the problem—seeing the situation for example as one of *protecting our national security* or of *preventing the construction of a Big Brother state*.

However, the best means of supporting or refuting particular ethical claims appears to be rigorous normative arguments. Unfortunately rigorous normative arguments are usually sufficiently complex, unintuitive, or contextually irrelevant, that they are largely ignored by citizens and by policymakers. Moreover, both politicians and ordinary citizens tend to be suspicious of the idea that bioethicists have any expertise to which they ought to defer, either in regards to bioethical methodology or in the substantive conclusions they reach. Insofar as theoretical bioethical claims do influence policy, the analysis that makes its way into policy will usually be markedly less sophisticated than what one would see in a high quality ethics journal, and will be deployed most often in the manner of means-end or specificatory reasoning, whereby the frame sets the end, and the argument draws out the implications of this framing for the issue at hand.

Existing political frames and narratives differ significantly between societies. A normative argument that is practically effective in one context, or in one community, will not necessarily gain policy traction in another. Thus, successfully pursuing the practical aim requires a much greater attention to context than successfully pursuing the theoretical aim. Montgomery (2013) provides a good example. The UK Organ Donation Taskforce, of which Montgomery was a member, decided not to recommend a change in policy from ‘opt in’ to ‘opt out’ for organ donation—despite seeing the potential rationale for an opt-out policy. One of the factors that swayed them against recommending an opt-out policy was the fact that the then Prime Minister, Gordon Brown, made public his support for such a policy. Unfortunately, Brown was at the time so unpopular that his support for the policy made it markedly more difficult for the Taskforce to recommend it. The Taskforce came to the conclusion that if they advocated an opt out policy, seemingly on the back of Brown’s views, it was likely that this would undermine the consensus they needed to achieve for many of their other recommendations to be successful. They therefore decided against recommending an opt-out policy.^2^

Pursuing the theoretical aim will typically involve making fine distinctions whose necessity will only become obvious once one has studied the area in depth. Research questions in academic bioethics often thus have (or can appear to have) a fractal quality: however much we zoom into a particular debate, there is still further detail to be looked at and further subtle distinctions to be made. Each distinction that is made provides the possibility for further disagreement, and so the net result is that academics naturally form themselves into a large number of camps, each with a very small number of people in them.^3^ Such fragmentation on the basis of small differences is an anathema to achieving the kind of political or systemic changes that are usually required by the practical aim. As political movements typically require an agreed platform that many people from a variety of different perspectives can sign up to, successful political arguments are usually not too specific and are nearly always significantly vaguer than fully articulated positions in theoretical bioethics are.^4^

Given this lack of congruence between the theoretical and the practical aims, it is natural to ask two questions. First, a question of scope: do both the practical and the theoretical aims fall within the scope of bioethics proper? (Of course, even if it were agreed that at least the core of both aims did fall inside bioethics proper, there would be room for further debate about whether there are elements of each that fall outside, and belong, say, to normative ethical theory on the one side or to political lobbying on the other side.) Second, assuming that at least a core of both the theoretical and the practical aims fall within bioethics proper, which should have the higher priority?

Questions such as these about the boundaries and purpose of disciplines embody only in a fairly superficial sense factual disputes about what most practitioners of the discipline would consider to be the boundaries of the discipline, and which types of questions they consider to be most important. In a deeper sense they are normative questions about how best to advance the discipline, considered as a a *tradition* in MacIntyre’s sense, as “an historically extended, socially embodied argument, and an argument precisely in part about the goods which constitute that tradition” (1981, p. 222).

Bioethics was transformed by one such set of arguments in the early years of this century, expanding from a discipline that examined primarily ‘Promethean’ new technologies and relationships between healthcare professionals and lay persons in wealthy countries, to one that is global in its outlook and focuses as much on institutions as individuals, and as much on the social determinants of health as on healthcare.^5^ What was determinative in this case was the sense that existing bioethics frameworks had overlooked (and were poorly equipped to deal with) large swathes of ethical questions connected to health and healthcare, including the social determinants of health and disease, the ethics of healthcare rationing, and the ethics of infectious disease.

This paper aims to start a different kind of reflexive conversation about the future of bioethics, focused not so much on which *topics* should be inside and which should be outside of bioethics, but about *why* we are doing bioethics, and what would count as doing it well. Just as in the case of the shift to a more institutional and global focus, the changes required are unlikely to follow clearly from a conceptual analysis of the idea of bioethics alone, or from taking a poll of what current individual bioethicists think that it is for, but by the gradual accretion of evidence of unsolved problems and missed opportunities, and a careful construction of a new and more fruitful framework. Hence, one of the main contributions of this article is to fashion an understanding of bioethics that allows us to see the theoretical and practical aims as interlinked and mutually supporting parts of a continuum rather than in antagonism to each other.

A few preliminary comments are in order before we turn to the constructive task. First, regardless of the stance that one takes on the relative importance of the theoretical versus the practical aims, it should be common ground that much work in bioethics is in fact motivated by a desire to mitigate or resolve ethical problems that have been made salient by changes in the lived environment. Perhaps a new procedure such as transplantation of mitochondrial DNA becomes available, which plausibly raises ethical issues that have not so far been considered; or it becomes apparent that antimicrobials are being (and have for some time been) used in an unsustainable way for some time, and many thousands will die unless the problem is brought under control.^6^ So whilst there may be reasons in other areas of philosophy for resisting the contamination of normative enquiry by practical concerns, and for framing normative enquiry as pure truth seeking, in bioethics at least, claiming only such a pure motivation would risk being disingenuous. Although the answers we seek might conceivably be timeless and discoverable through a priori reasoning alone, the questions that catch our attention are heavily influenced by our current concerns.

Second, whichever way one answers our two fundamental questions about bioethics, it should also be common ground that responding wisely to challenging ethical questions that arise from changes in the lived environment is an important *practical* goal. Even if we were to conclude that bioethics, properly considered, should have a purely theoretical aim, this would not make these pressing practical ethical problems go away; it would simply mean that we then needed another discipline or discourse that could translate between theoretical bioethics and these practical problems. We can call this the *translation problem.* Given that these practical ethical problems appear to be very significant, adequately addressing the translation problem would be important even if doing so fell outside of bioethics proper.

The last thing to say by way of preliminary is that there are a set of deep questions about the nature of ethics that bear on our two fundamental questions about bioethics—for example, whether we should view ethics as fundamentally a theoretical pursuit that aims at the discovery of normative truths, or whether we should think of ethics as a fundamentally practical endeavour that aims to solve various problems of coordination necessary for sustained and complex human life. I shall attempt to sidestep these deeper debates as far as is possible.^7^ My aim is to point out a set of problems that bioethics will need to solve regardless of whether we view ethics as fundamentally theoretical or fundamentally practical, and to provide a lens for thinking about these problems that will be attractive to thinkers from both perspectives. I shall argue that regardless of whether changing the world should form part of bioethics’s core aims, any plausible conception of bioethics should be at least committed to informing ethical judgements about what ought to be done in richly described real life cases. As much of the rest of the article explores, getting individual real world cases right is likely to require the kind of messy, complex and contextual work for which more abstract levels of theorising can only provide limited guidance. So, even if one’s orientation in bioethics is entirely towards the theoretical, questions about how to move between judgements based on simple cases and judgements in complex cases will be of central importance.

The rest of the paper proceeds as follows: Section 2 introduces various themes from debates on innovation in healthcare research—in particular debates around how to best connect up blue skies ‘basic’ research with practical innovations that can improve human lives—as a way of reflecting on the relationship between the theoretical and the practical aims in bioethics. It explains how a simple top-down linear model of innovation has been replaced by a more complex translational model, which recognises that innovations move along different pathways—both from the more ‘basic’ to the more ‘applied’ but also from the more ‘applied’ to the more ‘basic’. Section 3 adapts the translational model to bioethics—arguing that we should replace the idea of an opposition between the theoretical and the practical with the idea of a continuum from the most simple and abstract cases (thought experiments) to the most concrete and complex cases (real world cases). Insights need to travel in both directions along this continuum—both from the simpler and more abstract to the more complex *and* from the more complex to the simpler and more abstract. Section 4 examines some ways in which judgements that are correct in simple cases can fail to translate into helpful insights about more complex cases. Section 4.1 focuses on thought experiments, whilst Sect. 4.2 focuses on richer but still highly simplified cases. Section 5 concludes by reflecting on the implications of the analysis for the future of the discipline.

## The linear model in healthcare research

Questions about the relationship between theoretical advances and practical benefit are particularly urgent in healthcare research, owing to the ubiquity of death and suffering, the technical difficulty and expense of bringing new drugs to market, and governments’ own sizeable investments in funding both healthcare research and the successful treatments that result from it. So it is perhaps unsurprising that debate on the relationship between the theoretical and the practical is louder, older and more voluble in healthcare research than it is in bioethics, and contains a rich potential for cross-fertilisation. Moreover, approaching the question of the relationship between theory and practice in bioethics via debates about translational research in healthcare has the advantage that it allows us to sidestep the dispute between fundamentally theoretical and fundamentally practical approaches to ethics. By starting from outside ethics, in a domain in which it is relatively uncontroversial that there are theoretical facts that inquiry aims to discover, it becomes apparent that even in this domain, it follows neither that more abstract theoretical truths are more worthy of pursuit than more concrete ones, nor that inquiry should be guided by the pursuit of theoretical truths alone.^8^

World War II saw an unprecedented mobilisation of science for the war effort. The US set up the Office of Scientific Research and Development (OSRD) in 1941, whose goal was “to coordinate, supervise, and conduct scientific research on the problems underlying the development, production, and use of mechanisms and devices of warfare”, leading most notably to the Manhattan Project. As the war drew to a close, the US government faced a choice about the future of science funding: should it continue to funnel scientific research budgets towards short term solutions for centrally chosen goals, or would it be better to let scientists pursue their own endeavours without regard to practical application?

Vannevar Bush, the Director of OSRD, was tasked with answering this question. The resulting report, *Science: the endless frontier* set the tone for much of the approach to science funding over the next thirty years. Bush began from a distinction between basic and applied research:

Basic research is performed without thought of practical ends. It results in general knowledge and an understanding of nature and its laws. This general knowledge provides the means of answering a large number of important practical problems, though it may not give a complete specific answer to any one of them. The function of applied research is to provide such complete answers. (Bush 1945: Chapter 3.3)

Bush argued forcefully that it is the role of governments to support basic research, and that basic research is best undertaken in universities. Applied research, he argued, was better undertaken by businesses. It was crucial to his account that investing in basic science will (although in ways we cannot yet predict) have significant payoffs in the future:

One of the peculiarities of basic science is the variety of paths which lead to productive advance. Many of the most important discoveries have come as a result of experiments undertaken with very different purposes in mind. Statistically it is certain that important and highly useful discoveries will result from some fraction of the undertakings in basic science; but the results of any one particular investigation cannot be predicted with accuracy. (Bush 1945: Chapter 3.3)

The idea that theoretical work produces practical benefits—but in unexpected ways that it would be counterproductive to attempt to second-guess—is also at the heart of certain defences of philosophy that emphasise its long-term impact, but deny that this impact could usefully be measured or optimised in the short term.^9^ Of course, one obvious disanalogy is that, when compared to science, there is a decided lack of concerted action in either the commercial sector or in civil society to translate the results of basic normative or other philosophical work into commercialisable or socially beneficial results (though the use of game theory in management consultancy and elsewhere might be a possible analogue).

Bush’s approach to innovation was amplified and extended by a number of other figures over the next 20 years (as summarised for example, by Godin 2006 and Balconi et al. 2010), and the resulting approach to thinking about innovation has come to be known as the linear model. The linear model, at its most radical makes the following assumptions about how innovative products such as new drugs come to be invented:

(1.1) A clear distinction can be drawn between basic (scientific) and applied (technological and industrial) research… (1.2) Basic or fundamental or prior scientific research is the main or rather the unique source of technical innovation… (1.3) New knowledge acquired through basic research trickles down, almost automatically, to applied research, technology and innovations, even within short time spans. (Balconi et al. 2010, p. 5)

Each of these elements of the linear model has been criticised. First, as Stokes (1997) argues, scientific research projects cannot be divided neatly into those that aim to discover fundamental truths about the ways things are, and those that aim at practical application. Some forms of research aim both at practical applicability and at discovering fundamental laws. Louis Pasteur’s work, which aimed to address practical questions such as how to avoid milk and beer spoiling, but answered these questions by making fundamental discoveries such as the germ theory of disease, is a key exemplar of this (Stokes 1997, pp. 12–13).^10^

The idea that advances in basic science are always necessary for improved technologies—in Bush’s words, that “basic research is the pacemaker of technological progress,” (1945: Chapter 3.3) has also been challenged. Whilst there are obvious cases where new therapies have been developed out of a bedrock of advances in basic science (such as monoclonal antibodies), it is implausible to claim that healthcare innovation always starts from advances in basic science. Indeed, influential innovation scholars such as Kline and Rosenberg (1986, p. 288) argue that innovation led by basic research is the exception, rather than the rule.

Third, the idea that new basic science automatically leads to changes in medical practice that benefit patients is also highly questionable. The history of medical science is littered with examples of failures to properly connect basic and applied science. Even a case such as the discovery of penicillin, which might initially seem to be an obvious success story, shows how contingent takeup of basic science into applied science can be.^11^

As a result of the shortcomings of the linear model, a consensus has grown within healthcare research that funders and researchers need to think in systemic terms about how the different stages of research from the most basic science to the most applied can best fit together, an approach to thinking that has come to be known as translational research. As translational research developed, it became increasingly apparent that optimising the benefits to patients from research spending requires a rigorous and systematic focus on the pathways and transitions between the different stages of healthcare research, looking for bottlenecks and other opportunities to improve research efficiency.

## Bioethics and the translational research paradigm

Worries about the lack of direct connection between progress in basic theory and progress in answering more concrete questions apply even more strongly in bioethics than in healthcare research. As Section 1 explored, it is plausible to think that much high quality work in abstract normative theory has very little practical impact, and as we shall see, searching questions can also be asked about current efficiency in moving between ethical questions at different points of the continuum between the fully abstract and fully concrete.

In order to be able to talk sensibly about efficiency in translation between basic and applied contexts in a domain, we need at least a rough model of the continuum of different levels at which research questions can be asked and answered in that domain, and different potential ways of moving between them. Once we have such a model it is much easier to determine whether research insights are currently moving *between* these different levels as effectively as they could be.

Bioethicists do not often ask questions about research efficiency in their discipline, or explicitly address the question of how best to move between different levels of abstraction in ethical thinking, and so again the healthcare research literature will provide a starting-point. As Table 1 indicates, we can divide the journey from the most basic science to interventions that actually benefit patients into five stages.^12^ If an intervention that benefits patients is to be derived from basic science, it must pass through each of these stages.^13^ Given the uncontroversial focus in healthcare research prioritisation on the benefits to actual patients that are achieved at stage 5, the model immediately raises questions about research funding priorities if it turns out that prioritising basic science is a markedly less efficient way of achieving patient benefit than, say, research-led improvement of doctors’ prescribing habits.

**Table 1:** The translational continuum in healthcare research and in bioethics

Stage	Healthcare research stage	Bioethics equivalent
1	Basic science	Discussion of normative theory without any attempt to think about the applicability of moral theory to real life cases
2	Proof of concept—e.g. Phase 0 trial, testing pharmacodynamics and pharmacokinetics of drug to see that it could in principle be an effective therapy	Working out what ought to be done in thought experiments
3	Proof of efficacy—e.g. Phase 2 and 3 trials, demonstrating that the drug has a clinical effect under idealised conditions	Working out what ought to be done in simplified but somewhat realistic cases
4	Proof of effectiveness e.g. Pragmatic trial, to determine the effectiveness of the drug in real world clinical settings	Working out what we should do, all things considered, in real world situations
5	Implementation e.g. Policy changes, supported by healthcare research to benefit patients	Policy changes or other actions to make the world closer to how it should be

The model begins from the observation that much basic research—for example the discovery that a particular protein has a role in regulating inflammation—does not have obvious or immediate clinical application. It may take significant amounts of work to come up with a potential clinical application. The first main place in which there can be failures of translation is thus in moving from (1) basic science to (2) proof of concept in a potential clinical application. The journey from (2) proof of concept to (3) proof of efficacy, i.e. that the intervention works under idealised and controlled conditions, is long and arduous. There are many sites along this journey at which translation could potentially be improved, including better understanding of the limits of animal models, better research regulation, speedier marketing approval and so on. Once there is proof of efficacy, the next main stage is proof that the intervention will be safe and be more effective than its competitors in real world settings (4). However mere knowledge that an intervention is effective does not save lives by itself—so we can think of the final hurdle as the journey from proof of effectiveness (4) to patients being benefited by the intervention being adopted into standard clinical practice (5).

We can construct a roughly parallel trajectory for bioethics, tracing the kind of steps that would need to occur if new insights in basic normative theory were to lead to ethical improvements on the ground.^14^ As in the case of healthcare research, the idea is not to say that moral change *inevitably* follows this trajectory or that it should, but rather to allow us to use the model to ask questions about where time and other resources are currently being concentrated along this path, and whether transitions between different elements could be improved. As this is a first shot at constructing such a trajectory, it is likely that much could be improved.

First, we can take discussion of normative theory without any attempt to think about its applicability to be analogous to basic science. Normative theory of this kind will encompass questions such as what sorts of things are intrinsically valuable, the degree of commensurability of different intrinsic values, the range of different appropriate responses to things that are intrinsically valuable—whether all should be promoted for instance, or whether there are some that should be respected.^15^

Perhaps back in the early days of bioethics, it might have seemed plausible to think that we would be able to step straight from basic normative theory to claims about what should be done in the real world. But this possibility now seems fanciful.^16^ The continued deep and unresolved disagreements about matters of fundamental moral theory mean that even if it is clear what a particular moral theory would advise in a given case, knowing this does little to help us decide what to do when other undefeated moral theories recommend something else. Moreover, it has also become clear that moral theories lack the specificity to give us a definitive steer on many practical questions: as in the case of basic science, it will often take considerable work to develop a claim in fundamental normative theory to a point where it has clear implications for practice, even in idealised cases.

Thought experiments play a key bridging role in crossing the continuum between basic normative theory and judgements about real world cases. Thought experiments as I understand them here are toy philosophical cases that are designed to simplify a philosophical problem along a number of dimensions, thus making the problem more philosophically tractable. Like real-world experiments, their aim is to test a hypothesis, or to establish a point of principle—though the hypothesis or point of principle is an ethical one in the case of bioethical thought experiments. Thought experiments can be used to try to support or to undermine claims in basic normative theory, but they are also used as a controlled test environment for judgements about more complex and more realistic cases. As Frances Kamm explains it, “Real-life cases often do not contain the relevant—or solely the relevant—characteristics to help in our search for principles. If our aim is to discover the relative weight of, say, two factors, we should consider cases that involve only these two factors, perhaps artificially, rather than distract ourselves with other factors and options” (Kamm 1993, p. 7).

In the next section, I focus on some of the potential difficulties involved in moving from thought experiments to judgements about more complex cases, and from more complex cases to what should be done all things considered. However, it is important to notice that the main point of the model that I am proposing is that we need to think more rigorously and more systematically about how best to move between different levels of complexity in the analysis of ethical problems. Similar questions can and should be asked about moving from simpler to more complex cases on other parts of the continuum, about moving from complex cases to other cases of similar complexity, and about the implications of judgements about richly described complex cases for judgements about simpler and more abstract cases—points I return to in Sect. 5.

## Moving from simpler to more complex cases

### Internal and external validity in thought experiments

Thought experiments in bioethics are usually different in a number of salient aspects from the real world scenarios in which actual moral agents make their choices. Thought experiments in bioethics typically make all of simplifications 1–4, and may also make simplifications 5 and 6:

1. Authoritative ethical framing: the case raises the ethical question that the author of the thought experiment says it does. What the ethical issue is that the case raises is not subject to dispute.
2. Confined choices: choices must be made from a short predefined menu, with no ability to alter the terms of the problem. What counts as a viable response to the problem is stipulated by the author of the thought experiment, and is not up for discussion. The world of the thought experiment may operate according to laws that are plainly false of the real world. Hence, responses that would be likely to be effective in real world analogues of the thought experiment may be stipulated not to work, and other responses, which would be unlikely to be effective in real world analogues, may be stipulated to be effective.
3. Certainty of effect: each of the defined choices will bring about its stipulated effect with certainty. It is possible to identify in advance who will benefit, and who will lose, from each of the predefined choices. Where the choice is stipulated to bring about the desired effect a certain percentage of the time, these stipulated probabilities are entirely accurate.
4. Ceteris paribus: no morally relevant differences other than those which have been stipulated by the framer of the thought experiment apply to the situation.
5. Small numbers: the case is presented as one involving either single individuals, or groups of no more than five, even if the final desired aim is to make recommendations about situations involving large groups of people.
6. Atomicity: the case stands entirely on its own. There is no relevant history that affects judgements about the case, and no relevant future policy implications of making one decision rather than another.

Although the problem is not usually discussed in these terms, it is apparent that, just like clinical trials, the use of thought experiments can suffer from problems of internal and external validity. A clinical experiment is internally valid if it is designed in such a way that it correctly measures the causal effect of an independent variable or variables on one or more dependent variables. Doing so requires trial designers to avoid bias and confounding factors. Maintaining internal validity is the main reason for the shift towards randomised controlled trials. Similarly, a thought experiment is internally valid to the extent that it allows its readers to make judgements that are confident and free of bias about the hypothesis or point of principle that it aims to test. Thus for example, if the thought experiment requires comparing two cases which differ only in a single respect, we need to be confident that the cases *do* only differ in this one respect; and if the framer of the thought experiment claims that only one of the stipulated choices in the case is morally permissible, we should be able to judge confidently that this is true.

An experiment trial has external validity in addition to internal validity to the extent that the causal effects demonstrated in it can be generalised to a wide range of other situations. Within the fields of healthcare and public policy research, the relationship between internal and external validity has increasingly come into the spotlight. It is now a commonplace that an intervention that is efficacious in the highly idealised context of a controlled trial may not be effective in other contexts (Rothwell 2005). As Cartwright (2013) puts it, a randomised controlled trial (RCT) can show that an intervention worked *somewhere* but not that it will work *here.* To describe a trial as lacking in external validity is not by itself to impugn its quality as research: it is in the nature of an RCT that the rigour in experimental design that is necessary for internal validity sets limits on how, if at all, the results can be extrapolated to other contexts (Cartwright 2007). However, research that lacks external validity will be useful for changing the world only in a narrow range of contexts.

Similarly, we can say that a thought experiment has external validity in addition to internal validity to the extent that points of principle that are established by it can be generalised to a wide range of other circumstances. Thought experiments, and normative arguments more broadly, can lack external validity in at least two ways. First, if the principles they establish presuppose factual circumstances that are relevantly different from those that currently obtain. For example, Nozick’s entitlement theory of property rights is often thought to lack external validity on these grounds of factual irrelevance. The theory requires that property is justly held if and only if it was either (a) justly initially acquired or (b) legitimately transferred from someone else who was already entitled to it. Even leaving on one side the kinds of difficulties that can be raised to Nozick’s account of just acquisition, it is clear that there have been massive and systematic ruptures in justice in transfer throughout human history, including wars, colonialism, slavery, and the genocidal destruction of first nation peoples. Even if goods have been transferred legitimately since the time when they were unjustly acquired, Nozick’s view would not imply that they are now justly held. Rather such goods would be subject to a principle of justice in rectification—a principle that Nozick does not specify. So despite initial appearances, Nozick’s account tells us virtually nothing about who is entitled to what, or what could be legitimately taken from whom and redistributed to others, in our world with its particular history. To his credit, Nozick himself recognises the problem, and admits that his theory would require a full account of justice in rectification, which he is unable to offer, stating that “in the absence of such a treatment applied to a particular society, one *cannot* use the analysis and theory presented here to condemn any particular scheme of transfer payments, unless it is clear that no considerations of rectification of injustice could apply to justify it”^17^ Nozick (1975, p. 231).

Second, ethical principles and considerations are often claimed to interact with one another in holistic ways. On this view, there are scenarios in which ethical considerations that favour acting in certain ways in many or most cases no longer provide a reason in favour of acting in that way, and may even change polarity and provide a reason against acting in that way. For example, in usual circumstances, the fact that doing X will provide someone else with pleasure speaks in favour of it, but there are readily imaginable scenarios where the fact that something will create pleasure for someone would count against it (suppose the pleasure is sadistic). This view has the implication that even if we have an internally valid thought experiment, it would not follow that the principle established by the thought experiment will make the same contribution in other cases. So even if we have two precisely matched cases that differ only in one respect—say that one is a doing, and the other an allowing (as Rachels 1975 aims to do)—and establish that in this precisely controlled pair of cases, the factor that contrasts between the two cases does not make a moral difference, it would not follow that this factor will not make a moral difference in *other* cases.

The alternative to this holistic view seems to be to make the equally substantive and controversial assumption that each morally relevant factor makes its contribution to the overall ethical reasons in play independently from all other factors. Such a view, which Kagan (1988) calls the additive assumption, would (if true) provide a solid justification of the external validity of thought experiments in ethics. However, the fact that the additive assumption is itself highly controversial and has been subjected to many purported counterexamples, suggests that it would make work in bioethics less rather than more rigorous if bioethicists were to presuppose its truth in doing ethical reasoning.

Refusing to grant the additive assumption would place the external validity of thought experiments in ethics on a par with that of randomised clinical trials. In neither case would there be a logical entailment between internal validity and external validity. The failure of this logical entailment does not imply that randomised clinical trials cannot be externally valid, and would not imply that thought experiments in ethics cannot be externally valid. So to abandon the additive assumption is not say that the use of thought experiments should be (even could be) abandoned in bioethics, but rather to suggest that bioethicists need to be aware that even rigorously designed thought experiments may not show what their designers think they do and that consequently bioethicists need to be on the look out for, and to expect, defeaters that prevent translation from one context to another.^18^

One key question is whether there are some kinds of thought experiments that are more likely to lack external validity than others. Elster (2011) argues convincingly that outlandish thought experiments (involving, say, individuals with two-hundred arms) are much more likely to fail to be externally valid. In order to be confident that the thought experiment is externally valid, its readers need to be able to imagine in sufficient detail not only the outlandish elements of the thought experiment, but also the implications of the outlandish elements for ethical norms and practice within the world of the thought experiment. Given that thought experiments are usually very briefly described, there is much that can go wrong here: something that the author intended to be imaginable, such as a human being with arms so long they can reach from one end of India to the other^19^ may not really be really imaginable, or be imagined very differently by different readers.^20^ Even if a world containing such creatures is genuinely and stably imaginable, it may well be that taking the outlandish elements of the thought experiment seriously would entail such profound changes to other aspects of ethical life that there is little reason to think that ethical claims that are true of that world are transferable to our world.

### Ethical arguments and what should be done all things considered

Much work in bioethics analyses cases that are richer than thought experiments but still involve a number of important simplifications when compared to the real world. Presumably the hope is that whilst such cases are significantly simplified, it will nonetheless be reasonable to assume that the case discussed is realistic enough to have clear implications for what should be done all things considered in actual cases, and that the greater richness of the examples discussed will help to evade some of the problems of external validity that can affect thought experiments. Examples of this strategy would be examining the moral permissibility of an action type such as organ sales in the abstract, or analysing the validity of a particular argument against commercial surrogacy (say that it constitutes the selling of babies).

One worry about the external validity of this type of strategy, as I explore in more detail in Wilson (2009), is that it is much more difficult to justify conclusions about what ought to be done all things considered on the basis of philosophical analysis of arguments than some bioethicists seem to assume. For example, even if it could be adequately established that given valid consent and appropriate background conditions, sale of one’s organs is morally permissible, there is a long journey from this claim to the conclusion that therefore organ sale should be legalised. In scenarios closer to real life, the risks of exploitation, organ theft, crowding out of altruistic donation, transplant tourism or of black markets may be sufficiently high that we judge that it would not be justifiable to legalise organ sales, despite the fact that we can conceive of an idealised situation in which organ sales might be morally permissible.^21^

Focusing on the validity and soundness of ethical arguments is an indispensable tool for bioethicists. My claim is not that focusing on the quality of arguments is a bad way of doing bioethics, but that analyses of ethical arguments can be rigorous in the sense of attending carefully to the meaning of the terms and the pattern of logical inferences used, but yet still fail to be informative about what should be done all things considered. Just like thought experiments, more realistic cases can also suffer from problems of external validity.

## Conclusion: embracing complexity

We started from an apparent tension between the theoretical and the practical aims of bioethics, and two questions. First, a question about whether both the practical and the theoretical aims should fall within the scope of bioethics proper. Second, a question about which of the two aims should have higher priority. We are now in a position to answer and to reframe these questions.

The first question can be answered briefly. Normative theory without any thought to application (stage 1) is not a part of bioethics but of normative ethics more abstractly conceived. Any attempt to make normative judgements about cases (from thought experiments (stage 2) to real world cases (stage 4)) should uncontroversially be. Making correct (or at least well justified) moral judgements about cases becomes more complex the closer these cases approach to the real world. But this complexity seems no reason to draw the boundaries of bioethics in such a way that the questions it deals with are always simple. The world in which we act as moral agents is, after all, complex; and bioethics gains much of its practical impetus from the attempt to help provide normative guidance in this world. So there is little reason to think that the boundaries of bioethics should stop short of including making wise judgements about complex individual cases.

Once one has determined what is ethically required all things considered in response to a real world situation, and if one has at least some power to influence the situation, it would be odd to think that this had no implications for what one should do. I thus find it difficult to deny that if a bioethicist comes to a particular conclusion about a real world case on the basis of a careful analysis of the relevant reasons, and then seeks to change the world on the basis of this analysis (by means such as arguing for the view publicly) that they do what they do as a bioethicist. So, at least where the means used are rational persuasion, stage 5 is very plausibly a core part of bioethics.

On the second question, we can now see that the supposed opposition between the theoretical and the practical aims of bioethics from which we started is misleading. Theoretical questions in ethics form a continuum from the simplest questions such as whether knowledge or pleasure is valuable in itself, to the most complex, such as what ought to be done, and by whom, to combat the rise of antimicrobial resistance.^22^ Working out what ethical duties are held and by whom in a real world case is still a theoretical question, and like the simplest questions about cases involving one value in isolation, requires us to bring together relevant theoretical claims with situational judgement. The main difference is that as we travel across the continuum in one way, more and more of the messiness and complexity of the real world is bracketed for simplicity, and in the other way, more and more of the messiness and complexity of the real world is revealed.

Hence, even focusing entirely on bioethics as a theoretical rather than a practical pursuit gives no reason in itself to think that the examination of simpler and more abstract cases is more important than messier and more complex ones. A further argument would be needed for this claim. The key question could be rephrased as whether the messiness and complexity of the real world is something that stands in the way of us getting a grip on what is really going on, ethically speaking, or whether this messiness and complexity *is* what is going on, ethically speaking. Many philosophers assume, implicitly if not explicitly, that the former view is correct.^23^ This assumption makes it seem natural that, other things being equal, work on simpler and more abstract problems will be more fruitful for the discipline, given that such work appears to give rise to principles that will apply to a wider variety of cases, whilst analyses of richly described real world cases seem to tell us only about that particular case.

We have seen that, given the implausibility of the linear model, things are likely to be more complicated than this. Principles established through apparently rigorous abstract work may fail to have external validity. Analysis of rich cases can themselves have important theoretical implications: for example, they may bring out problems or contradictions that went unnoticed in a more abstract model. Analysis of real world cases can also be more widely useful to the practice of bioethics through the power of example: seeing how complex and contested empirical material bears on what should be done in one ethical problem may be useful in determining how complex and contested empirical material bears on a different ethical problem.^24^ As in the case of healthcare research, it seems plausible to assume that determining which are the better, and which the worse, ways of moving between simpler and more complex research problems is itself a complex research problem—a problem that this paper has introduced, but not solved.

What follows from this analysis for the future of bioethics? Perhaps the main lesson is that work in bioethics can fail to be rigorous in two distinct ways. In explaining this I made use of the concepts of internal and external validity. First, work in bioethics can be shoddy on its own terms: thought experiments can be poorly equalised, distinctions made inexactly, arguments vague or fallacious. Second, work that is rigorous on its own terms can be unhelpful, or misleading, if authors extrapolate its lessons to other cases without due attention to the factors that could undermine external validity. Bioethics journals are full of attempts to do research that is rigorous in the first sense, and to expose cases where others have done research that is not rigorous in this sense. But as yet there is very little written on, and little attention to, rigour in the second sense. If bioethics is going provide a useful basis for improving the world, this needs to change.

Two brief suggestions for the future. First, bioethicists should be clearer and more explicit about the implications (if any) that they think that their arguments about simpler cases have for other more complex cases. They should not make claims that their analysis has policy implications unless they are willing for those policy implications to be taken seriously.^25^ Second, bioethics needs a literature on how best to move between different levels of complexity, both on what counts as good practice in this area, and what is required to train the next generation of bioethicists. Over to you.

## Footnotes

1 For accessible introductions to some of the literature that supports this claim, see for example Lakoff (1996), Westen (2008).

2 The point here is not that the Taskforce made the right decision, but the more general one that there can be a significant gap between what ethical arguments, considered acontextually, would seem to recommend, and what is the best thing to do all things considered in a particular richly described context.

3 On this point, see the useful discussion of the “narcissism of minor difference” in Wolff (2012, pp. 1–6).

4 Montgomery (2013) explores some of these issues in greater depth. He argues that we should distinguish between academic bioethics and ‘public ethics’, with public ethics being the branch that aims to bring about political and regulatory change.

5 For more on this turn, see e.g. Daniels (2006). I took some steps towards constructing a new framework for thinking about public health ethics in Wilson (2009, 2011).

6 On mitochondrial DNA see e.g. Nuffield Council on Bioethics (2012), and on antimicrobial resistance see e.g. Millar (2011).

7 It is perhaps worth remarking that it is not very clear what could count as non question-begging evidence for the claim that ethics is fundamentally a theoretical as opposed to a practical endeavour (or vice versa).

8 Philip Kitcher has used similar thoughts to argue for an approach to priority-setting in science that he calls “well-ordered science” (Kitcher 2001, 2011). Briefly, the argument is that there are an infinity of possible theoretical problems that researchers could be working on at any one time, and however many problems they solve, there will always be an infinity remaining. Given this, it makes sense to think that there should be some kinds of norms to guide enquiry, above and beyond merely seeking truths or well-justified beliefs. Where there are an infinity of truths to be discovered, it no longer seems adequate to simply say “I’m seeking to discover truths”. All human beings could do that all day every day until the end of time, and it would still remain the case that there were an infinity of unknown truths. As Dennett (2006) explores, it is thus quite possible for large amounts of intellectual labour to be expended on problems that whilst genuine, are not good uses of the limited resources that we have to devote to inquiry.

9 See for example, British Philosophical Association (2009), Bovens and Cartwright (2010).

10 It is a little harder to see what an ethics example of something that fell into what Stokes describes as "Pasteur’s Quadrant" would be like: what would work look like that succeeded in clarifying simultaneously a fundamental normative or theoretical concept, and which had an important practical implication in the way that we live our lives? One possibility might be Parfit’s (1984) discussion of the non-identity problem; another might be Glover’s (1975) refutation of the claim that it is only harms that are noticeable which matter morally.

11 Fleming discovered the antibiotic properties of penicillin by accident in 1928 in the course of his basic research. He published the discovery, and even went as far as to use it to treat a colleague’s conjunctivitis, but was unable to interest his colleagues in it. (Fleming 1929; Le Fanu 2011). Finding little takeup for the idea of penicillin’s therapeutic use, and having failed to find a way of concentrating it, he abandoned the project. (Ligon 2004) It was only ten years later that Florey and Chain chanced on Fleming’s paper, and undertook an animal trial, which showed penicillin to be effective in mice, and the potential benefit for human life became obviously apparent.

12 The translational research literature contains a number of different conceptualisations of this continuum. For an overview of this debate, and a nuanced response to it, see Drolet and Lorenzi (2011).

13 Of course (and acknowledging this was key to the shift away from the linear model), innovations that benefit patients can also have rather different trajectories. Healthcare innovations need not travel “from the bench to the bedside”, but may instead travel “from the bedside to the bench” (Marincola 2003). Other important innovations can come from organising things better *within* a given stage of research (for example by reducing administrative delays in setting up clinical trials).

14 My thoughts on the relationship between theory and practice in ethics have been enriched by a number of conversations over the years with Alan Cribb (see Cribb 2010, 2011). Cribb (2010) floated the idea of a translational ethics, but treated it merely as a “heuristic device”, to facilitate reflection on the broader project of “translating ethical scholarship into policy or practice” (2010, p. 207). This paper takes the idea of translational ethics seriously and more literally as a future path for bioethics.

15 For an overview of some of these debates, see Schroeder (2012).

16 On this point, see for example Caplan (1983), and the critique of the “engineering model” of applied ethics therein.

17 Nozick’s argument presents a particularly clear case of a factual failure of external validity—but it is a problem to which theories such as luck egalitarianism are also notoriously vulnerable.

18 Thanks for Simon Rippon for pushing me to be clearer on this point.

19 See Kamm (2006, p. 352) for this thought experiment: “Suppose that I stand in a part of India, but I have very long arms that reach all the way to the other end of India, allowing me to reach a child who is drowning in a pond at a great distance (Reach Case). Intuitively, I think that this case is treated as one where the child is near.”

20 It seems from the context that Kamm (2006, p. 352) intends that the extra-long arms be an integrated part of the individual's body. This raises a number of challenges for imagining the case: is the rest of the person supposed to be scaled up in proportion to their thousand-mile long arms? The context suggests not. But if we are supposed to imagine an ordinary-sized human being with arms a thousand miles long, how is their anatomy supposed to work? How much would thousand-mile long arms weigh? How would the weight be supported? How would it be possible for them to perform everyday tasks such as dressing themselves, taking a shower, or eating a sandwich?

21 For a nuanced account, which attempts to examine these factors and more, see Cohen (2013).

22 On the complexity of AMR as an ethical problem, see for example Littmann (2014).

23 See for example, GA Cohen's demand that the subject matter of political philosophy be the search for fact-free fundamental principles of justice: “facts are irrelevant in the determination of fundamental principles of justice. Facts of human nature and human society of course (1) make a difference to what justice tells us to do in specific terms; they also (2) tell us how much justice we can get; and they (3) bear on how much we should compromise with justice, but, so I believe, they make no difference to the very nature of justice itself.” (2008, p. 285).

24 For a good example of this approach in action, see Thacher (2006), who uses rich empirical work on city design and grassroots political activism to show how such concrete work can reveal values, and value conflicts, that would otherwise be missed.

25 Confusion about this was one of the problems with Giubilini and Minerva's (2013) now notorious paper on “after-birth abortion”. After causing a storm of protest by claiming that “‘after-birth abortion’ (killing a newborn) should be permissible in all the cases where abortion is, including cases where the newborn is not disabled,” (2013, p. 261) the authors then were forced to clarify that “we never meant to suggest that after-birth abortion should become legal,” (Giubilini and Minerva 2012) and had been interested only in making philosophical arguments.

## References

[CR1] Balconi M, Brusoni S, Orsenigo L (2010). In defence of the linear model: An essay. Research Policy.

[CR2] Bovens, L., and Cartwright, N. 2010. Measuring the impact of philosophy. Science and Technology Parliamentary Committee Papers on Research Funding Cuts. http://www.publications.parliament.uk/pa/cm200910/cmselect/cmsctech/memo/spendingcuts/uc8302.htm.

[CR3] British Philosophical Association. 2009. Impact in the research excellence framework. http://www.bpa.ac.uk/resources/uploads/2009/11/bpa-impact-position-paper-301109.pdf.

[CR4] Bush V (1945). Science: The endless frontier.

[CR5] Caplan AL (1983). Can applied ethics be effective in health care and should it strive to be?. Ethics.

[CR6] Cartwright N (2007). Are RCTs the gold standard?. BioSocieties.

[CR7] Cartwright N (2013). Knowing what we are talking about: Why evidence doesn’t always travel. Evidence & Policy: A Journal of Research, Debate and Practice.

[CR8] Cohen GA (2008). Rescuing justice and equality.

[CR9] Cohen I (2013). Transplant tourism: The ethics and regulation of international markets for organs. The Journal of Law, Medicine & Ethics.

[CR44] Cribb, A. 2010. Translational ethics? The theory–practice gap in medical ethics. *Journal of medical ethics* 36(4): 207–210. 10.1136/jme.2009.029785 20338930

[CR45] Cribb, A. 2011. Beyond the classroom wall: Theorist-practitioner relationships and extra-mural ethics. *Ethical theory and moral practice* 14(4): 383–396.

[CR10] Daniels N (2006). Equity and population health: Toward a broader bioethics agenda. The Hastings Center Report.

[CR11] Drolet B, Lorenzi N (2011). Translational research: Understanding the continuum from bench to bedside. Translational Research.

[CR12] Dennett DC (2006). Higher-order truths about chmess. Topoi.

[CR46] Elster, J. 2011. How outlandish can imaginary cases be? *Journal of applied philosophy* 28(3): 241–258.

[CR13] Fleming A (1929). On the antibacterial action of cultures of a penicillium, with special reference to their use in the isolation of B. influenzae. British Journal of Experimental Pathology.

[CR14] Giubilini, A., and Minerva, F. 2012. An open letter from Giubilini and Minerva. http://blogs.bmj.com/medical-ethics/2012/03/02/an-open-letter-from-giubilini-and-minerva/.

[CR15] Giubilini A, Minerva F (2013). After-birth abortion: Why should the baby live?. Journal of Medical Ethics.

[CR16] Glover J (1975). It makes no difference whether or not I do it. Proceedings of the Aristotelian Society.

[CR17] Godin B (2006). The linear model of innovation: The historical construction of an analytical framework. Science, Technology and Human Values.

[CR18] Kagan S (1988). The additive fallacy. Ethics.

[CR19] Kamm F (1993). Morality, mortality volume I: Death and whom to save from it.

[CR20] Kamm F (2006). Intricate ethics.

[CR21] Kitcher P (2001). Science, truth and democracy.

[CR43] Kitcher, P. 2011. Philosophy inside out. *Metaphilosophy* 42(3): 248–260

[CR22] Kline S, Rosenberg N, Landau R, Rosenberg N (1986). An overview of innovation. The positive sum strategy: Harnessing technology for economic growth.

[CR23] Lakoff G (1996). Moral politics: What conservatives know that liberals don’t.

[CR24] Le Fanu J (2011). The rise and fall of modern medicine.

[CR25] Ligon B (2004). Penicillin: Its discovery and early development. Seminars in Pediatric Infectious Diseases.

[CR26] Littmann, J. (2014). *Antimicrobial resistance and distributive justice*. PhD thesis, UCL.

[CR27] MacIntyre AC (1981). After virtue: A study in moral theory.

[CR28] Marincola FM (2003). Translational medicine: A two-way road. Journal of Translational Medicine.

[CR29] Millar M (2011). Can antibiotic use be both just and sustainable… or only more or less so?. Journal of Medical Ethics.

[CR30] Montgomery J (2013). Reflections on the nature of public ethics. Cambridge Quarterly of Healthcare Ethics.

[CR31] Nozick R (1975). Anarchy, state and Utopia.

[CR32] Nuffield Council on Bioethics (2012). Novel techniques for the prevention of mitochondrial DNA disorders: An ethical review.

[CR33] Parfit D (1984). Reasons and persons.

[CR34] Rachels J (1975). Active and passive euthanasia. The New England Journal of Medicine.

[CR35] Rothwell P (2005). External validity of randomised controlled trials: “To whom do the results of this trial apply?”. The Lancet.

[CR36] Schroeder, M. 2012. Value theory, the Stanford encyclopedia of philosophy (Summer 2012 Edition). In ed. Edward N. Zalta. http://plato.stanford.edu/archives/sum2012/entries/value-theory/.

[CR37] Stokes DE (1997). Pasteur’s quadrant: Basic science and technological innovation.

[CR38] Thacher D (2006). The normative case study. American Journal of Sociology.

[CR39] Westen D (2008). Political brain: The role of emotion in deciding the fate of the nation.

[CR40] Wilson J (2009). Towards a normative framework for public health ethics and policy. Public Health Ethics.

[CR41] Wilson J (2011). Why it’s time to stop worrying about paternalism in health policy. Public Health Ethics.

[CR42] Wolff J (2012). Ethics and public policy: A philosophical inquiry.

